# Vaginal discharge during pregnancy and associated adverse maternal and perinatal outcomes

**DOI:** 10.12669/pjms.37.5.4187

**Published:** 2021

**Authors:** Meharunnissa Khaskheli, Shahla Baloch, Aneela Sheeba Baloch, Syed Ghulam Sarwar Shah

**Affiliations:** 1 Meharunnissa Khaskheli, MBBS, FCPS. Department of Obstetrics and Gynecology, Liaquat University of Medical and Health Sciences, Liaquat University Hospital, Jamshoro, Hyderabad, Sindh, Pakistan; 2 Shahla Baloch, MBBS, DGO, FCPS. Department of Obstetrics and Gynecology, Liaquat University of Medical and Health Sciences, Liaquat University Hospital, Jamshoro, Hyderabad, Sindh, Pakistan; 3 Aneela Sheeba Baloch, MBBS, DMRD, FCPS. Department of Radiology Liaquat University of Medical and Health Sciences, Liaquat University Hospital, Jamshoro, Hyderabad, Sindh, Pakistan; 4 Syed Ghulam Sarwar Shah, MBBS, MSc, PhD. NIHR Oxford Biomedical Research Centre, Oxford University Hospitals NHS Foundation Trust, John Radcliffe Hospital, Headington Way, Headington, Oxford, OX3 9DU, England, UK. Radcliffe Department of Medicine, University of Oxford, John Radcliffe Hospital, Headington Way, Headington, Oxford, OX3 9DU, England, UK

**Keywords:** Feto-maternal complications, Pregnancy outcomes, Pregnant women, Vaginal discharge

## Abstract

**Objectives:**

To observe the effects of vaginal discharge during pregnancy on maternal and fetal outcomes.

**Methods:**

This observational study was undertaken form June 2018 to 31 May 2019 period in the Department of Obstetrics and Gynaecology at Liaquat University of Medical and Health Sciences hospital Jamshoro Unit IV. Data were collected from a convenience sample of 85 pregnant women. All the pregnant women with vaginal discharge were included in the study, while the women with bleeding and other medical disorders during pregnancy were excluded. Data was analyzed.

**Results:**

Women’s mean age as 27.4 (±4.7) years and most were 28-35 weeks pregnant (n=29, 34%) and primigravida (n=35, 41%). Seventy six women (89%) presented with vaginal discharge while nine women (11%) reported no vaginal discharge. Of those with vaginal discharge,53 women (69.7%) had vaginal infections: bacterial vaginosis (n=21, 39.6%), vaginal candidiasis (n=17, 32.1%) and vaginal trichomoniasis (n=15, 28.3%). Pathological vaginal discharge (PVD) was associated with vaginal irritation (n=30, p<0.0001), vaginal pain (n=50, p<0.0001), fever (n=12, p=0.015), uterine contractions (n=31, p<0.0001), premature membrane rupture (n=29, p<0.0001), abortion (n=13, p=0.009), pre-term delivery (n=24, p<0.0001) and post-partum endometritis (n=19, p=0.0006). PVD was associated with neonatal outcomes i.e. low birth weight (n=24, p<0.0001), low Apgar score at birth (n=22, p=0.0001), neonatal respiratory distress syndrome (n=21, p=0.0002), neonatal intensive care hospitalisation (n=20, p=0.002) and early neonatal death (n=16, p=0.003).

**Conclusion:**

Pathological vaginal discharge (PVD) during pregnancy is more frequent and is associated with adverse maternal and perinatal outcomes.

## INTRODUCTION

Vaginal discharge (VD) is a frequent gynaecological complaint in women during their reproductive life and especially during pregnancy.^1^,^2^ Women however could not generally differentiate between normal (physiological) and abnormal (pathological) vaginal discharge.^3^ Proper diagnosis of VD requires clinical examination and laboratory investigations.^4^ Normal vaginal discharge, also known as leucorrhoea, is thin, clear or milky white fluid with a mild odor. It is one of the earliest signs of pregnancy and it progressively increases in amount and continues throughout the pregnancy whereas pathological vaginal discharge (PVD) varies in color from dirty white to yellowish green and it may be foul smelling. PVD commonly results due to vaginal infections such as bacterial vaginosis, trichomonas vaginalis and vaginal candidiasis or candidial vaginitis, which is also known as vulvovaginal candidiasis.^2^

Bacterial vaginosis (BV) is a common vaginal infection caused by the imbalance of microorganisms of vulvovaginal area. Although mostly asymptomatic, BV increases risk of sexually transmitted infections (STI), HIV/AIDS, trichomonas vaginalis and vaginal candidiasis.^5^,^6^ It is also associated with adverse pregnancy outcomes i.e., premature rupture of membranes, preterm labor and delivery, and postpartum endometritis. Vaginal candidiasis (VC) is the commonest fungal infection of vagina, which is caused by the overgrowth of candida species.^7^ These are the flora that normally present in the lower genital tract in healthy and asymptomatic women.^8^ In pregnant women, candida albicans is the most common type of vaginal candida species followed by candida glabrata.VC is commonly associated with VD, which is whitish in color. The rate of VC is higher in women treated with broad spectrum antibiotics and those who are pregnant, diabetic and immune suppressed such as HIV/AIDS patients. Vaginal candidiasis is associated with vaginal pain, difficult or painful sexual intercourse and vulvovaginal irritation and itching. It is also associated with pregnancy complications such as abortion, premature birth, and low birth weight.

Trichomonas vaginalis (TV) is a parasitic infection of vagina caused by the flagellated parasitic protozoan called Trichomonas vaginalis. It affects women in their reproductive life and pregnancy and it is the most common non-viral STI globally. Symptoms of TV in women include VD that is often yellow-green, diffuse and malodorous.TV is associated with dysuria, itching, vulvar irritation, abdominal pain, pelvic inflammatory disease and poor birth outcomes e.g., premature rupture of membranes, preterm delivery and low birth weight.^9^

BV, VC and TV affect about 6% women.^10^ All of these infections are associated with VD and may lead to vaginal dysbiosis (abnormal vaginal microbiota) during early stages of pregnancy which is commonly associated with adverse pregnancy outcomes.^11^ About half of pregnant women with PVD experience pruritus (itching), malodour (very unpleasant smell), dysuria (painful or difficult urination), and dyspareunia (difficult or painful sexual intercourse) In addition, VD is associated with psychological and mental disorders.^12^ Vaginal infections and associated VD are more prevalent in women of low literacy poor socio-economic background and living in lower and middles income countries such as Pakistan. Pathological vaginal discharge, especially in pregnant women, is an important public health issue due to its association not only with socio-psychological impacts during pregnancy but also due to its association with adverse maternal and foetal outcomes as mentioned above. There is therefore need for further research on VD in pregnant women especially those of lower socio-income background and living in lower and middle income countries such as Pakistan where there is a higher prevalence of vaginal candidiasis and bacterial vaginosis during pregnancy.^13^ The primary objective of this study was to differentiate between normal (physiological) and abnormal (pathological) VD during pregnancy. The secondary objective was to study whether PVD was associated with adverse maternal and perinatal outcomes.

## METHODS

This observational study was conducted in Department of Obstetrics and Gynaecology at Liaquat University of Medical and Health Sciences hospital Jamshoro Unit IV. All pregnant women with complaint of VD were invited to the study. All pregnant women with threatened abortion, cervical incompetence, placental abnormalities, history of (Sexually transmitted infection) STI and treatment at STI clinics were excluded from the study. The subjected study population were recruited by convenience sampling of 85 pregnant women from June 1st 2018 to 31st May 2019. All women were recruited after taking their informed written consent. This study was approved (Ref: LUMHS/REC/-768, Dated: 25-05-2018) by the research ethics committee of university. These women had regular antenatal visits, delivered in the hospital and were followed in the period of puerperium. They were evaluated thoroughly by taking detailed history, clinical examination and investigations. For every woman, three samples of VD were taken for laboratory investigations. The first sample was stored in a vial with 95% alcohol for cytological examination that sample was taken from squamocolumnar junction of cervix to exclude cervical pathology like CIN as those who had perception for vaginal discharge can have cause of discharge related to cervix; the second sample was utilized in the amine test; and the third sample was taken with a cotton swab, which was immersed in normal saline (1 ml, in sterile glass) for direct examination. The samples were sent to Pathological Diagnostic Research Centre for pathological examination and report. For candidiasis and trichomoniasis, direct microscopically examination was taken as the gold standard for seeing hyphae and flagellate. For diagnosis of bacterial vaginosis, the Amsel criteria were taken as the gold standard. The diagnosis of bacterial vaginosis was confirmed by the presence of three of following four conditions i.e., vaginal discharge, vaginal pH>4.5, positive result in the amine test and presence of clue cells on microscopy. These women were given treatment according to the cause.

Study variables, included participant’s age, parity, gestation period, vaginal discharge, associated complaints of vaginal irritation and pain, smell, true onset of labor pains as well as presence of any existing maternal, pregnancy and neonatal problems.

Data was collected on predesigned proforma and analyzed by frequencies and descriptive statistics for continuous variables while categorical variables were analyzed with Chi Square tests for statistically significant differences between the observed and expected frequencies between two or more categories. P-value ≤0.05 was considered as statistically significant. There were no missing values. Data were analyzed in the SPSS, version 23 for Windows (IBM Corp 2015).

## RESULTS

Demographic and gestation characteristics: In total, 85 pregnant women who took part in this study. Women’s age varied between 20 and 37 years (mean age = 27.4 ±4.7 years). Majority of women were 26-31 years old (n=34, 40%), 28-35 weeks pregnant (n=29, 34%) and primigravida (n=35, 41%) (Table-I).

**Table-I:** Participants’ demographic and gestational characteristics (N=85).

				Vaginal discharge		

			No	Yes		P value

	Number	Percentage		Pathological	Physiological	
Age						0.228
20-25 years	32	37.7	3	17	12	
26-31 years	34	40.0	5	24	5	
32-37 years	19	22.3	1	11	7	
Gestational period						0.124
8-14 weeks	15	17.6	2	5	8	
15-21 weeks	17	20	3	9	5	
22-27 weeks	24	28.2	3	16	5	
28-35 weeks	29	34.1	1	22	6	
Parity						0.566
Primigravida	35	41.2	4	20	11	
Para 2-4	34	40.0	5	21	8	
Para 5 and above	16	18.8	0	11	5	

Out of 85 pregnant women who presented with vaginal discharge, seventy-six women (89%) had VD on clinical examination, while nine women (11%) had no obvious vaginal discharge. Laboratory examination of VD samples confirmed presence of vaginal infections in 53 women i.e., bacterial vaginosis (n=21, 39.6%), candidiasis (n=17, 32.1%), and trichomoniasis (n=15, 28.3%), these cases were declared as PVD (Table-II). No growth of any pathogenic organism was found in the remaining 23 samples, which were declared as normal VD (Table-II). Normal VD was watery in color (n=23,100%), a slight stain in the quantity (n=22, 95.65%) and odorless (n=23, 100%) whereas pathological discharge was mostly yellowish curd like in color (n=17, 32.1%), soaking clothes in the quantity (n=35, 66%) and foul smelling (n=28, 52.8%) Compared to women with normal vaginal discharge, a higher proportion of women with PVD was statistically significantly associated with vaginal irritation (n=30, p<0.0001), vaginal pain (n=50, p<0.0001), fever (n=12, p=0.015) (Table-II).

**Table-II:** Vaginal discharge its types, characteristics, associated symptoms and laboratory investigation reports (N=85).

		Vaginal discharge		

Vaginal discharge		No obvious discharge only perception	Yes	

			Normal physiological discharge	Pathological discharge

		Count (%)	Count (%)	Count (%)

		9 (10.6)	23(27.05)	53(62.35)
***Physical characteristics***				
***Colour***				
No discharge		9 (10.6)	0	0
Watery		0	23 (27.1)	11 (12.9)
Yellowish curd like		0	0	17 (20)
Frothy		0	0	12 (14.1)
Muddy		0	0	13 (15.3)
***Quantity***				
No discharge		9 (10.6)	0	0
Slight stain		0	22 (25.9)	6 (7.0)
Soaking clothes		0	1 (1.2)	35 (41.2)
Copious in amount		0	0	12 (14.1)
***Odour***				
No discharge		9 (10.6)	0	0
Odourless		0	23 (27.1)	11 (12.9)
Fishy odour		0	0	14 (16.5)
Foul smelling		0	0	28 (32.9)
***Associated Symptoms***				
***Vaginal Irritation***				
a. Yes	31 (36.5)	0	1 (1.17)	30 (35.29)
b. No	54 (63.5)	9 (10.6)	22 (25.88)	23 (27.05)
***Vaginal pain***				
a. Yes	56 (65.88)	2(2.35)	4 (4.70)	50 (58.82)
b. No	29 (34.11)	7(8.23)	19 (22.35)	3 (3.52)
***Fever***				
a. Yes	12 (14.1)	0	0	12 (14.11)
b. No	73 (85.9)	9 (10.6)	23 (27.05)	41 (48.23)
***Laboratory investigation / Pathogenic organism***				
Absent		9 (10.6)	23 (27.1)	0
Present		0	0	53 (62.3)
Bacterial Vaginosis		0	0	21 (24.7)
Vaginal Candidiasis		0	0	17 (20.0)
Vaginal Trichomoniasis		0	0	15 (17.6)
Cervical cytology report for screening CIN		Negative	Negative	Negative

Compared to women with normal vaginal discharge, a higher proportion of women with PVD was statistically had significant rate of preterm uterine contractions (n=31, p<0.0001), premature membrane rupture (n=29, p<0.0001), miscarriage (n=13, p=0.009), pre-term delivery (n=24, p<0.0001) and post-partum endometritis (n=18, p<0.0001) (Table-III). PVD was also statistically significantly associated with adverse neonatal outcomes: low birth weight (n=24, p<0.0001), low Apgar score at birth (n=22, p=0.0001), neonatal respiratory distress syndrome (n=21, p<0.0001), neonatal intensive care hospitalization (n=21, p,0.0001) and early neonatal death (n=16, p<0.0001) (Table-III).

**Table-III:** Adverse Maternal and Perinatal Outcomes Associated with Vaginal discharge during Pregnancy (N=85).

Maternal Outcomes	Count	Percentage	Vaginal Discharge			P value

			No discharge only perception	Yes		

				Pathological	Physiological	
Miscarriage						<0.009
No	72	84.7	9	40	23	
Yes	13	15.3	0	13	0	
Uterine contractions						<0.0001
No	41	56.94	9	9	23	
Yes	31	43.05	0	31	0	
Premature rupture of membranes						<0.0001
No	43	59.72	9	11	23	
Yes	29	40.27	0	29	0	
Preterm delivery						<0.0001
No	48	66.7	9	16	23	
Yes	24	33.3	0	24	0	
Postpartum endometritis						<0.0001
No	54	75	9	22	23	
Yes	18	25	0	18	0	
Perinatal Outcomes Low birth weigh						<0.0001
No	48	66.7	9	16	23	
Yes	24	33.3	0	24	0	
Low Apgar^†^ Score at birth						<0.0001
No	50	69.4	9	18	23	
Yes	22	30.6	0	22	0	
Neonatal respiratory distress syndrome						<0.0001
No	51	70.8	9	19	23	
Yes	21	29.2	0	21	0	
Neonatal intensive care hospitalization						<0.0001
No	51	70.8	9	20	22	
Yes	21	29.2	0	20	1	
Early neonatal death						<0.0001
No	56	77.78	9	24	23	
Yes	16	22.22	0	16	0	

† Apgar =Appearance, Pulse, Grimace, Activity, and Respiration.

## DISCUSSION

During pregnancy VD is common. Normal VD was not a major issue for women while pathological VD was a serious problem due to infection its symptomatology such as colour, quantity and odour parameters and associated complications.^14^

### Causes of pathological vaginal discharge

In this study it was found that VD in pregnancy was most commonly pathological and it was due to three types of vaginal infections i.e. bacterial vaginosis, vaginal candidiasis and trichomoniasis vaginalis same is reported by other studies.^15^,^16^ The result of this study showed that bacterial vaginosis was the most common vaginal infection followed by vaginal candidiasis while trichomoniasis vaginalis was the least common vaginal infection in pregnant women with VD this is in consistent with other study.^17^

These vaginal infections are common in pregnant women with low level of education, underprivileged social and economic status and poor reproductive health hygiene.^18^ This study was conducted in public sector hospital outpatient Obstetrics and Gynaecology clinics where vast majority of patients attending these outpatient services belong to poor families, living in rural areas and slums, and often with low education. In addition, the average literacy rate of female in the country is low (52%), which is even lower in rural areas (40.5%). These factors are associated with low reproductive health hygiene in women.^19^ Moreover, females in Pakistan have less knowledge and practice of health hygiene and healthcare during menstruation, pregnancy, delivery and the postpartum period. More importantly, women living in rural areas of Pakistan have low healthcare seeking behavior during pregnancy, which is determined by social, economic and cultural factors.^20^-^24^

These factors may contribute to pathogenic vaginal infections i.e. BV, VC and TV and resultant PVD during pregnancy as identified in this study and in prior research.^25^ These infections and VD need proper investigation and appropriate treatment at the earliest to avoid any possible perinatal complications that may be associated with these vaginal infections.^26^,^27^ However there were no statistically significant differences detected in the preponderance of normal and PVD during pregnancy based on the women’s age, gestation period and parity this is in consistent with other study.^28^

It was observed from the findings that in a higher proportion of pregnant women PVD was associated with adverse maternal and perinatal outcomes. The adverse maternal outcomes associated with PVD were vaginal irritation and pain, uterine contractions, premature membrane rupture, abortion, pre-term delivery and post-partum endometritis.^29^ The adverse perinatal outcomes significantly associated with PVD were low birth weight, low Apgar score at birth, neonatal respiratory distress syndrome, neonatal intensive care hospitalization and early neonatal death.^25^ These findings provide empirical evidence that PVD in pregnant women is associated with adverse perinatal outcomes this is in consistence with other study.^29^ PVD in pregnant women needs clinical investigation and relevant treatment.^15^ Proper management of vaginal infections and PVD during pregnancy may help in reducing the risk of adverse perinatal outcomes.^29^ that are very common in low middle income countries(LMICs) such as Pakistan.^30^

## CONCLUSION

VD is common during pregnancy and it is essential to differentiate between normal VD and pathological VD during pregnancy. pathological VD is commonly due to vaginal infections such as bacterial vaginosis, candidiasis and trichomoniasis, which can be treated with appropriate treatment and avoided through information and advice for improving general health and reproductive health hygiene. In addition, early examination of vaginal discharge in pregnancy is needed to check for pathological VD to avoid associated adverse maternal and perinatal outcomes. Therefore, all the pregnant women with vaginal discharge should be investigated and treated appropriately.

### Authors’ Contribution:

**MK:** Conception, design, study conducted, Manuscript writing.

**SB:** Acquisition of data, Manuscript review.

**ASB:** Data collection and Interpretation.

**SGSS:** Analyses of data & Drafting and critically revised.
